# cDNA Cloning, Overexpression, Purification and Pharmacologic Evaluation for Anticancer Activity of Ribosomal Protein L23A Gene (*RPL23A*) from the Giant Panda

**DOI:** 10.3390/ijms13022133

**Published:** 2012-02-16

**Authors:** Bing Sun, Yi-Ling Hou, Wan-Ru Hou, Si-Nan Zhang, Xiang Ding, Xiu-Lan Su

**Affiliations:** Key Laboratory of Southwest China Wildlife Resources Conservation (Ministry of Education), College of Life Science, China West Normal University, 637009, Nanchong, China; E-Mails: 13890878727@163.com (B.S.); starthlh@126.com (Y.-L.H.); zsn_520520@163.com (S.-N.Z.); dingxiang319@yahoo.cn (X.D.); 15228111295@163.com (X.-L.S.)

**Keywords:** Giant Panda, RPL23A, cDNA, cloning, overexpression, purification, anticancer activity

## Abstract

*RPL23A* gene encodes a ribosomal protein that is a component of the 60S subunit. The protein belongs to the L23P family of ribosomal proteins, which is located in the cytoplasm. The purpose of this paper was to explore the structure and anti-cancer function of ribosomal protein *L23A* (*RPL23A*) gene of the Giant Panda (*Ailuropoda melanoleuca*). The cDNA of *RPL23A* was cloned successfully from the Giant Panda using RT-PCR technology. We constructed a recombinant expression vector containing *RPL23A* cDNA and over-expressed it in *Escherichia coli* using pET28a plasmids. The expression product obtained was purified by using Ni chelating affinity chromatography. Recombinant protein of *RPL23A* obtained from the experiment acted on Hep-2 cells and human HepG-2 cells, then the growth inhibitory effect of these cells was observed by MTT (3-[4,5-dimethyl-2-thiazolyl]-2,5-diphenyl-2H-tetrazolium bromide) assay. The result indicated that the length of the fragment cloned is 506 bp, and it contains an open-reading frame (ORF) of 471 bp encoding 156 amino acids. Primary structure analysis revealed that the molecular weight of the putative RPL23A protein is 17.719 kDa with a theoretical pI 11.16. The molecular weight of the recombinant protein RPL23A is 21.265 kDa with a theoretical pI 10.57. The *RPL23A* gene can be really expressed in *E. coli* and the RPL23A protein, fusioned with the N-terminally His-tagged protein, gave rise to the accumulation of an expected 22 KDa polypeptide. The data showed that the recombinant protein RPL23A had a time- and dose-dependency on the cell growth inhibition rate. The data also indicated that the effect at low concentrations was better than at high concentrations on Hep-2 cells, and that the concentration of 0.185 μg/mL had the best rate of growth inhibition of 36.31%. All results of the experiment revealed that the recombinant protein RPL23A exhibited anti-cancer function on the Hep-2 cells. The study provides a scientific basis and aids orientation for the research and development of cancer protein drugs as well as possible anti-cancer mechanisms. Further research is on going to determine the bioactive principle(s) of recombinant protein RPL23A responsible for its anticancer activity.

## 1. Introduction

Ribosomes, the organelles that catalyze protein synthesis, consist of a small 40S subunit and a large 60S subunit. Together these subunits are composed of 4 RNA species and approximately 80 structurally distinct proteins [1,2]. The *RPL23A* gene encodes a ribosomal protein that is a component of the 60S subunit. The protein belongs to the L23P family of ribosomal proteins. It is located in the cytoplasm. As is typical for genes encoding ribosomal proteins, there are multiple processed pseudogenes of this gene dispersed through the genome [3].

Ribosomal protein gene mutations or disturbance in their expression levels have been found in many inherited genetic diseases such as Diamond-Blackfan anaemia syndrome, Tuner syndrome, Noonan syndrome, Camurati-Engelmann disease, and BardetBiedl syndrome 4. In recent years, along with progress in science technique with unremitting and continuous work going deep into research, the physiological function in the human disease of the ribosomal protein has been progressively clarified [4]. Some achievements show that the *RPL23A* gene has been playing a role in cell growth and regulation. A component of ribosomal proteins, human ribosomal protein L23A (RPL23A), identified as mda20, is one of the genes down-regulated in human melanoma and other cell types treated with IFN-b. So the RPL23A protein may be one of the target molecules involved in mediating growth inhibition by interferon [5]. In yeast, the corresponding protein binds to a specific site on the 26S rRNA. The *RPL23A* gene is co-transcribed with the U42A, U42B, U101A, and U101B small nucleolar RNA genes, which are located in its third, first, second, and fourth introns, respectively [6]. Another study found that the ribosomal proteins L23A can each be imported alternatively by any of the importinβ, transportin, RanBP5 and RanBP7 receptors. RPL23A has been studied in detail and a very basic region to which each of the four import receptors bind avidly has been identified. This domain might be considered as an archetypal import signal that evolved before import receptors diverged in evolution [7].

Cancer is one of the leading causes of mortality worldwide. Cancer, known medically as a malignant neoplasm, is a term for a large group of different diseases, all involving unregulated cell growth [8]. In cancer, cells divide and grow uncontrollably, forming malignant tumors, and invade neighbouring parts of the body. The cancer may also spread to more distant parts of the body through the lymphatic system or bloodstream [9]. Cancer is one of the most fatal diseases in the human population and one of the most frequent causes of death worldwide [10,11]. Many proteins possess antitumor activity. The present study was carried out to evaluate the anti-cancer activity of Ribosomal Protein L23A (RPL23A) from the Giant Panda.

At present, the sequence information for *RPL23A* gene has been reported in other fields [5,6,12]. However, *RPL23A* gene from the Giant Panda (*A. melanoleuca*) has not been reported, especially in aspect of anti-cancer function. The Giant Panda (*A. melanoleuca*) is one of the oldest and rarest species, currently found only in China, and known as the “National treasure of China”. It belongs to the most endangered species in the world. In the past years, scientists have achieved tremendous success with the macrograph of the Giant Panda. Recently, functional gene study became one of the hottest issues in current Giant Panda research [13–20]. Now we are paying more attention to ribosomal protein RPL23A of the Giant Panda because of its many biological functions especially in relation to anticancer activity.

In this study, according to the related information for ribosomal protein *L23A* gene (*RPL23A*) of the designed primer of some mammalians, including *Homo sapiens*, *Mus musculus*, *Rattus norvegicus* and *Macaca mulatta*, we used RT-PCR technique to amplify the cDNA of *RPL23A* gene from the total RNA of the skeleton muscle of the Giant Panda. We then analyzed the sequence characteristics of the protein encoded by the cDNA and compared it with those of human and other animals reported. We constructed a recombinant expression vector contained *RPL23A* cDNA and over-expressed it in *E. coli* using pET28a plasmids. Under the optimized expression conditions, we obtained a lot of recombinant protein of RPL23A from the Giant Panda, which then was purified using Ni chelating affinity chromatography. Recombinant protein obtained from the experiment acted on human laryngeal carcinoma Hep-2 cells and human hepatoma HepG2 cells, then displayed a different cell growth depressive effect. The present study was carried out to evaluate the anti-cancer activity of ribosomal protein L23A (RPL23A) from the Giant panda on human laryngeal carcinoma Hep-2 cells and human hepatoma HepG2 cells. The purpose is to reveal the anti-cancer activity of ribosomal protein L23A (RPL23A) from the Giant panda. The study will provide scientific data and means for postulating the anti-cancer mechanism, for pharmaceutical research and for the development of the gene from the Giant Panda.

## 2. Results and Discussion

### 2.1. Analysis of the cDNA of RPL23A from the Giant Panda

**Figure 1:**
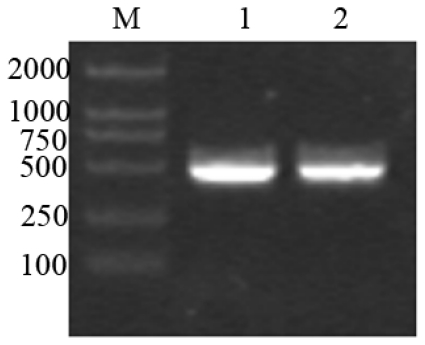
Reverse transcription polymerase chain reaction products of the Giant Panda *RPL23A*. M: Molecular Marker DL2000; 1, 2: amplified *RPL23A*.

About 500 bp of cDNA fragment was amplified from the Giant Panda. The length of the cDNA cloned was 506 bp (Figure 1).

**Figure 2:**
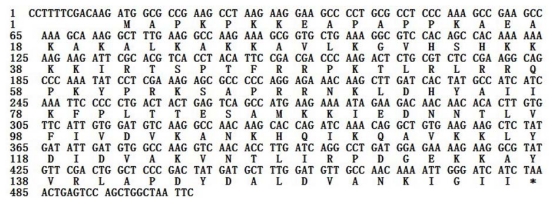
Nucleotide and deduced amino acid sequences of cDNA encoding the Giant Panda *RPL23A.* The asterisk (*) represents stop codon.

On the basis of the high identity, we concluded that the cDNA isolated is the cDNA encoding the Giant Panda RPL23A protein. The *RPL23A* sequence was submitted to Genbank (accession number: HQ318070). The 506 bp of the Giant Panda *RPL23A* sequence contains a 13 bp 5′-untranslated sequence and a 22 bp 3′-unstranslated region. An open-reading frame (ORF) of 471 bp encoding 156 amino acids was found in the cDNA sequence (Figure 2).

Alignment analysis of *RPL23A* of the Giant Panda and those of *Homo sapiens*, *Mus musculus*, *Rattus norvegicus* and *Macaca mulatta*, indicated that both the nucleotide sequence and the deduced amino acid sequence are highly conserved. There is no deletion or insertion of nucleotide and amino acid residue. As determined by BLAST analysis, the nucleotide sequence RPL23A cloned from the Giant Panda shares a high homology with those of *Homo sapiens*, *Mus musculus*, *Macaca mulatta* and *Rattus norvegicus* of 90.02%, 88.96%, 91.30% and 88.32%, respectively; the homologies for amino acid sequences are all 99.87% compared with the four species. This striking pattern of evolutionary conservation is considered reasonable, as ribosomal protein genes are a group of highly conserved housekeeping genes [21].

### 2.2. Prediction and Analysis of Primary Structure, Protein Functional Sites and Advanced Structure in RPL23A Protein of the Giant Panda

Primary structure analysis revealed that the molecular weight of the putative RPL23A protein of the Giant Panda is 17.7179 kDa with a theoretical pI 11.16 (Table 1). Like most ribosomal proteins, L23A is highly basic, containing a combined 45 Arg, Lys, and His residues and only 14 Asp and Glu residues, in which the highest content is the Lys residues (19.23%), far higher than other amino acids and without Cyc and Trp residues. Physical and chemical analysis showed that the molecular weight of the putative protein among the five mammalians is very close and the theoretical pI is identical (Table 1). The secondary structure analysis of RPL23A protein indicated that the protein was 15.38% in E (strand), 44.87% in H (helix) and 39.74% in C (coil). The secondary structure is slightly different compared with *Homo sapiens*, *Macaca mulatta*, *Mus musculus* and *Rattus norvegicus* (Table 2).

**Figure 3:**
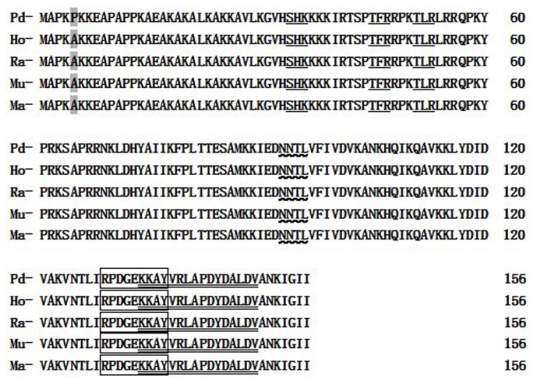
Comparison of the RPL23A amino acid sequences among the different species. 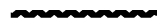: N-glycosylation site; 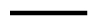: Protein kinase C phosphorylation site; □: Tyrosine kinase phosphorylation site; 
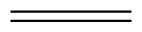
: Ribosomal protein L23 signature; 
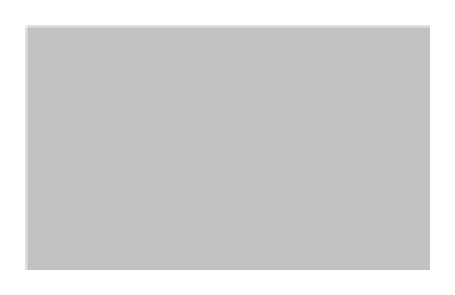
: polymorphism site. (Pa: *A. melanoleuca*; Ho: *Homo sapiens; Mu: Mus musculus*; Ra: *Rattus norvegicus*; Ma: *Macaca mulatta*).

Topology prediction shows that there is one N-glycosylation site, one Tyrosine kinase phosphorylation site, three Protein kinase C phosphorylation sites and one Ribosomal protein L23 signature in the RPL23A protein of the Giant Panda (*A. melanoleuca*) (Figure 3). Further analysis detected only one polymorphic site in the amino acid sequences of the five species compared (Figure 3). Site 5 is a Pro in the Giant Panda, but the other mammals have an Ala at this site. This polymorphism of C-to-G and T-to-G synonymous mutation, were detected at the first and third base of codon 5 (CCT→GCG, Pro→Ala) in the Giant Panda. But this polymorphic site is not located in the functional site, so it does not result in any differences between the Giant Panda and the other four species. Also, most base transitions of the gene coding sequence in these mammals were synonymous mutations. These synonymous mutations did not result in any changes in the corresponding DNA information and did not change the amino acid in the expression product. Consequently the spatial structure of the corresponding protein is not affected.

### 2.3. Overexpression and Purification of the RPL23A Gene in *E. coli*

**Figure 4:**
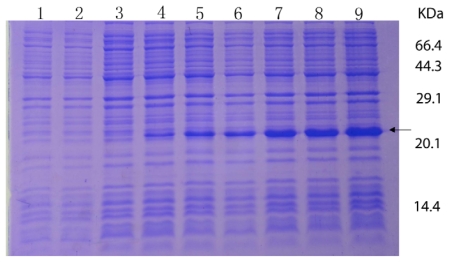
Protein extracted from recombinant *E. coli* strains were analyzed by SDS-PAGE gel stained with Commassie blue R 250. Numbers on right shows the molecular weight, and the arrow indicates the recombinant protein bands induced by IPTG with 0, 0.5, 1, 1.5, 2, 2.5, 3 and 3.5 h (lane 2–9), respectively. The lane 1 represents the products of the *E. coli* strains with the empty vectors.

The *RPL23A* gene was overexpressed in *E. coli*, using pET28a plasmids carrying strong promoter and terminator sequences derived from phage T7. For this purpose, the *RPL23A* gene was amplified individually by PCR and cloned in a pET28a plasmid, resulting in a gene fusion coding for a protein bearing a His-tag extension at the N terminus. Expression was tested by SDS-PAGE analysis of protein extracts from recombinant in *E. coli* BL21 strains (Figure 4). The results indicated that the protein *RPL23A* fusion with the N-terminally His-tagged form gave rise to the accumulation of an expected 22 kDa polypeptide that formed inclusion bodies. Apparently, the recombinant protein was expressed after half an hour of induction and after 3.5 h reached the highest level. The expression product obtained could be used for purification and further study of its function.

**Figure 5:**
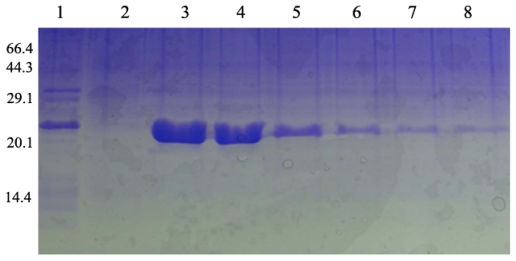
The purification of recombinant proteins RPL23A (Lane 1: RPL23A proteins extracted from recombinant *E. coli*; Lane 2: effluent liquid collected from columns; Lane 3–8: the eluent collected from columns).

Under the optimized expression conditions, we obtained a lot of recombinant protein, which then was purified by using Ni-NTA chelating affinity chromatography. SDS-PAGE analysis clearly indicated that there are about 22 kDa polypeptides in the third and fourth lanes (Figure 5). Protein separation and purification are key steps in genetic engineering technology. Through affinity chromatography, we obtained purified protein. During affinity chromatography, the protein solution PH was changed twice which enabled us to achieve a high purity of protein. It means that the protein solution firstly goes through the column under acid conditions, and then finally outflows from the column by changing the PH of the effluent liquid. SDS-PAGE analysis clearly indicated that there are about 22 kDa polypeptides in the third and fourth lane. Size consistency of the purified protein and unpurified RPL23A protein suggest that the protein is only the protein encoded by the RPL23A from the Giant Panda. The sequence of acquired ribosomal protein RPL23A gene recombinant protein from the Giant Panda consists of 190 amino acid residues. The molecular weight of recombinant protein RPL23A was 21.265 kDa and the PI was 10.57.

### 2.4. Anticancer Activity of Ribosomal Protein L23A (RPL23A) from the Giant Panda on Human Laryngeal Carcinoma Hep-2 cells and Human Hepatoma HepG2 Cells

Laryngeal cancer may also be called cancer of the larynx or laryngeal carcinoma. Most laryngeal cancers are squamous cell carcinomas, reflecting their origin from the squamous cells which form the majority of the laryngeal epithelium. Laryngeal cancer may spread by direct extension to adjacent structures, by metastasis to regional cervical lymph nodes, or more distantly, through the blood stream. Distant metastates to the lung are most common [22]. A hardy cell line, HEp-2 resists temperature, nutritional, and environmental changes without a loss of viability. It has supported the growth of 10 of 14 arboviruses as well as the measles virus, and it has been used for experimental studies of tumor production in rats, hamsters, mice, embryonated eggs and volunteer terminal cancer patients [23,24]. HepG2 (Hepatocellular carcinoma, human) cells are epithelial in morphology, and secrete a variety of major plasma proteins; e.g., albumin, transferrin and the acute phase proteins fibrinogen, alpha 2-macroglobulin, alpha 1-antitrypsin, transferrin and plasminogen [25]. Our experiments gave different results because of the differences between these two kinds of cancer cells.

**Figure 6:**
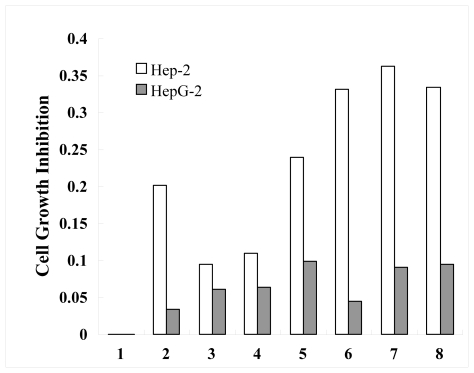
Cell growth inhibition of Ribosomal Protein L23A (RPL23A) from Giant Panda on human laryngeal carcinoma Hep-2 cells and human hepatoma HepG-2 cells (1–8: The concentration of recombinant protein RPL23A of 0, 7.4, 2.96, 1.48, 0.74, 0.37, 0.185, 0.148 μg/mL, respectively).

To assess whether the recombinant protein RPL23A demonstrates anticancer activity in human laryngeal carcinoma Hep-2 cells and human hepatoma HepG2 cells, these cells were then cultured in the presence and absence of various concentrations of the RPL23A for 24 h. The human laryngeal carcinoma Hep-2 cells and human hepatoma HepG2 cells treated with 0.148–7.4 μg/mL of RPL23A for 24 h displayed different cell growth inhibition when assayed using MTT compared to the control (untreated) cells. For comparison, human hepatoma HepG-2 cells displayed no significant change when compared to the control (untreated) cells, while the RPL23A has a time and dose dependent on Hep-2 cell growth inhibition. The data indicate that the effect at low concentrations is better than at high concentrations, and the concentration of 0.185 μg/mL has the best rate of growth inhibition, which is 36.31% (Figure 6). The proliferation of human laryngeal carcinoma hep-2 cells was thus restrained by recombinant protein RPL23A. The principle may be that there are some RPL23A receptors on the face of the human laryngeal carcinoma hep-2 cells, which combine with recombinant protein RPL23A. Further studies of the mechanism and the signal transduction pathways are in progress.

### 2.5. The effect of Ribosomal Protein L23A (RPL23A) from the Giant Panda on Morphology of Human Laryngeal Carcinoma Hep-2 cells

**Figure 7:**
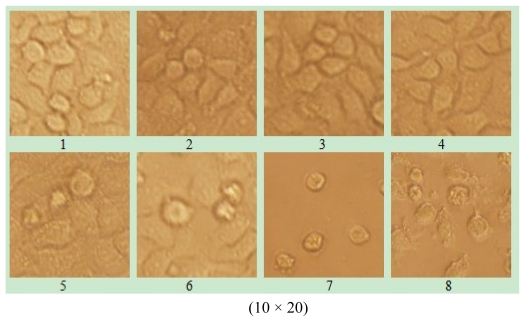
The effect of Ribosomal Protein L23A (RPL23A) from the Giant Panda on morphology of human laryngeal carcinoma Hep-2 cells for 0, 7.4, 2.96, 1.48, 0.74, 0.37, 0.185, 0.148 μg/mL (pictures 1–8), respectively.

The 96-well plates were placed under inverted microscope; the camera records different concentration changes for cell morphology in order to measure the effect of recombinant protein RPL23A. RPL23A exhibited high anticancer activity as can be seen from the cell morphology which was rounded into groups, and even cracked off in pieces in the Hep-2 group, while Hep G-2 cells displayed no significant change compared to those in the control group (data not shown) (Figure 7).

## 3. Experimental Section

### 3.1. Materials

Skeletal muscle was collected from a dead Giant Panda at the Wolong Conservation Center of the Giant Panda (Sichuan, China). The collected skeletal muscle was frozen in liquid nitrogen and then used for RNA isolation. The human laryngeal carcinoma Hep-2 cells were bought in the biochemistry and molecular immune research institute of North Sichuan Medical College. Total Tissue/cell RNA Extraction Kits were purchased from Waton Company (Shanghai, China). Reverse transcription kits were from Promega Company (Beijing, China). Gel Extraction Mini Kits were purchased from OMEGA Corporation (Shanghai, China). PMD-18 T Vector Systems and restriction enzyme BamHI and HindIII were got from TaKaRa Bio Group (Dalian, China). DNA polymerases were purchased from Sangon Co (Shanghai, China). Host bacteria *E. coli* DH5α were stored in Key Laboratory of Southwest China Wildlife Resources Conservation. CW0009 Ni-Agarose His-tag Protein purification kits were purchased from Ealysino Biological Technology Co. (Beijing, China). Bradford Protein Assay Kits were purchased from Majorbio Biotech Co. (Shanghai, China).Penicillin/streptomycin (penicillin 10,000 units/mL, streptomycin 10,000 μg/mL) and Dulbecco’s minimal essential medium (DMEM) reagents were purchased from Gibco BRL (Grand Island, NY, USA). Fetal bovine serum was obtained from Sijiqing Co. (Guangzhou, China).

### 3.2. RNA Isolation

Total RNAs were isolated from about 600mg of muscle tissue using the Total Tissue/Cell RNA Extraction Kits (Waton Inc., Shanghai, China) according to the manufacturer’s instructions. The total RNAs extracted were dissolved in DEPC (diethypyrocarbonate) water, and kept at −70 °C.

### 3.3. Primers Design, RT-PCR

The PCR primers were designed by Primer Premier 5.0, based on the mRNA sequence of *RPL23A* from *Homo sapiens* (NM_000984), *Mus musculus* (NM_207523), *Rattus norvegicus* (NM_001108283) and *Macaca mulatta* (NM_001193565). The specific primers of cDNA sequence are as follows:

Pd*-RPL23A*-F: 5′-CCTTTTCGACAAGATGGCG-3′;

Pd*-RPL23A*-R: 5′-GAATTAGCCAGCTGGACTCA-3′.

Total RNAs were synthesized into the first-stranded cDNAs using a reverse transcription kit with Oligo dT as the primers, according to the manufacturer’s instructions (Promega, Beijing, China). The 20 μL of first-strand cDNA synthesis reaction system included 1 μg of total RNAs, 5 mM of MgCl_2_, 1 mM of dNTPs, 0.5 μg of Oligo dT_15_, 10 U/μL of RNase inhibitor, and 15U of AMV reverse transcriptase, and was incubated at 42 °C for 60 min.

The first-strand cDNA synthesized was used as a template. The total reaction volume for DNA amplification was 25 μL. Reaction mixtures contained 1.5 mM of MgCl_2_, 200 μM of each of dATP, dGTP, dCTP and dTTP (Omega, Kanpur, India), 0.3 μM of each primer, 5.0 units of Taq plus DNA polymerase (Sangon Co., Shanghai, China). DNA amplification was performed using a MJ Research thermocycler, Model PTC-200 (Watertown, MA, USA) with a program of 4 min at 94.0 °C, followed by 30 cycles of 1 min at 94.0 °C, 0.5 min at 46 °C and 1.5 min at 72.0 °C, and then ended with the final extension for 10 min at 72.0 °C. After amplification, PCR products were separated by electrophoresis in 1.5% agarose gel with 1× TAE (Tris-acetate-EDTA) buffer, stained with ethidium bromide and visualized under UV light. The expected fragments of PCR products were harvested and purified from gel using a DNA harvesting kit (Omega, Kanpur, India), and stored at −20°C.

### 3.4. Cloning and Identifying the cDNA Sequence

The harvested PCR products were ligated into a pMD19-T vector at 4 °C for 12 h. The recombinant molecules were transformed into *E. coli* complete cells (DH5α), and then spread on the LB-plate containing 50 μg/mL ampicillin, 200 mg/mL IPTG (isopropyl-beta-D-thiogalactopyranoside), and 20 mg/mL X-gal. Plasmid DNA was isolated and digested by *Pst*I and *Sca*II to verify the insert size. Plasmid DNA was sequenced by Huada Zhongsheng Scientific Corporation (Beijing, China).

### 3.5. Construction of the Expression Vector and Overexpression of Recombinant RPL23A

PCR fragment corresponding to the RPL23A polypeptide was amplified from the *RPL23A* cDNA clone with the forward primer, 5′-CAGAATTCATGGCGCCGAAG C-3′ (EcoRI) and reverse primer, 5′-CGGTCGACTTAGATGATCCCA-3′ (SalI), respectively. The PCR was performed at 94 °C for 3 min; 35 cycles of 30 s at 94 °C, 45 s at 53 °C and 1 min at 72 °C; 10 min at 72 °C. The amplified PCR product was cut and ligated into the corresponding site of pET28a vector (Stratagen). The resulting construct was transformed into *E.coli* BL21 (DE3) strain (Novagen) and used for the induction by adding IPTG (isopropyl-b-D-thiogalactopyranoside) at an OD600 of 0.6 and culturing further for 4 h at 37 °C, using the empty vector transformed BL21 (DE3) as a control. The recombinant protein samples were induced after 0, 0.5, 1, 1.5, 2, 2.5, 3 and 3.5 h and then separated by SDS-PAGE and stained with Commassie blue R 250.

### 3.6. Purification of Recombinant Protein RPL23A

Acquired genetic engineering recombinant protein has a tag which is comprised of six histidine (His-tag), so nickel chelating affinity chromatography is available as follows. Centrifuge the ultrasonic product to collect sediments, and then suspend them with Soluble Binding Buffer (20 mM Tris-HCl, 0.5 M NaCl, 10 mM imidazole, pH 7.9) until the inclusion body is clean. Centrifuge and suspend the sediments with Inclusion Body Binding Buffer (20 mM Tris-HCl, 0.5 M NaCl, 5 mM imidazole, urea 8 M, pH 7.9) in the ice, until the inclusion body is thoroughly dissolved. Centrifuge and transfer the above supernatant to the chromatography column with nickel. Stand for 2 min after the above supernatant is completely transferred so that the six His-tag and nickel in the padding can combine fully. Inclusion Body Binding Buffer of 15 times the column volume is used to flush the column, so as to wash the uncombined protein. Collect the excurrent liquid. Then, inclusion Body Elution Buffer of 5 times the column volume is used to wash the combined protein. According to the amount of the column volume, collect the outflow liquid. Then, SDS-PAGE is used to detect the effect of purification. The concentration of recombinant protein is determined by Bradford Protein Assay Kits. To further purify the elution protein, dialysis is available. After 48 h of desalination, the purified protein with 10% glycerol is stored at −20 °C.

### 3.7. Cell Lines Culture

Human laryngeal carcinoma Hep-2 cells and human hepatoma HepG-2 cells were purchased from North Sichuan Medical College, Institute of Biochemistry and Molecular Immunology and maintained for study in MEM (Gibco, Carlsbad, CA, USA) supplemented with 10% fetal bovine serum (FBS, Evergreen biological Products company, Shanghai, China), 100 U/mL penicillin (Gibco Company, Billings, USA), 100 μg/mL streptomycin (Gibco Company, Billings, USA), pH 7.4 RPMI-1640(Gibco Company, Billings, USA) Cells were cultured in a 5% CO_2_/95% air incubator at 37 °C.

### 3.8. Test the Cell Viability of Purified Recombinant Protein RPL23A on Human Laryngeal Carcinoma Hep-2 Cells and Human Hepatoma HepG-2 Cell Activity by MTT Assay

These cells were seeded into 96-well microculture plates at appropriate densities to maintain the cells in an exponential phase of growth throughout the duration of the experiment. Human laryngeal carcinoma Hep-2 and human hepatoma HepG-2 cells were exposed to RPL23A protein at 0, 7.4, 2.96, 1.48, 0.74, 0.37, 0.185, 0.148 μg/mL for 24 h and each concentration was evaluated in six separate wells, respectively. At the end of the exposure, 10 μL of MTT was added to each well and the plates were incubated for 4 h at 37 °C. Then, 150 μL of DMSO was added to each well and the plates were surged for 5 min. The optical density (OD) was read on a plate reader (BIO-RAD Co., Beijing, China) at two wavelengths of 490 nm. Media-alone as well as control wells, in which PMBE was absent, were included in all experiments. The degree of inhibition of cell proliferation was calculated using the following formula: Growth inhibition (%) = (OD control − OD treated)/OD control × 100%.

### 3.9. Data Analysis

The sequence data were analyzed by GenScan software (http://genes.mit.edu/GENSCAN.html). Homology research of the Giant Panda *RPL23A* compared with the gene sequences of other species was performed using Blast 2.1 (http://www.ncbi.nlm.nih.gov/blast/). ORF of the DNA sequence was searched using ORF finder software (http://www.ncbi.nlm.nih.gov/gorf/gorf.html). The values of WM and pI were computed using the Compute pI/Mw tool (http://www.expasy.org/tools/pi_tool.html). Protein structure of the RPL23A sequence cloned was analyzed using PredictProtein software (http://cubic.Bioc.columbia.edu/predictprotein/). Multiple Sequence Alignment was performed by software DNAstar Lasergene and DNAMAN 6.0.

## 4. Conclusions

The results indicated that the length of fragment cloned is 506 bp, and that it contains an open-reading frame (ORF) of 471 bp encoding 156 amino acids. Primary structure analysis revealed that the molecular weight of the putative RPL23A protein is 17.719 kDa with a theoretical pI 11.16. The molecular weight of the recombinant protein RPL23A is 21.265 kDa with a theoretical pI 10.57. The *RPL23A* gene can be really expressed in *E. coli* and the RPL23A protein, fusioned with the N-terminally His-tagged protein, gave rise to the accumulation of an expected 22 KDa polypeptide. The data showed that the recombinant protein RPL23A had a time- and dose-dependency on the cell growth inhibition rate. The data also indicated that the effect at low concentrations was better than at high concentrations on Hep-2 cells, and the concentration of 0.185 μg/mL had the best rate of growth inhibition of 36.31%. For comparison, human hepatoma HepG-2 cells displayed no significant change when compared to the control (untreated) cells.

In recent years, more and more research has indicated that the high level expression of ribosomal protein is a prognostic factor in some kinds of tumor, and several ribosomal proteins have been identified in high levels in cancers [26–30]. Our research showed that ribosomal protein L23A can effectively inhibit human laryngeal carcinoma Hep-2 cells growth or proliferation activity. The findings will give scientific support and orientation for research and development of anticancer protein drugs of ribosomal protein L23A recombinant protein and for the development of tumor vaccine for tumor prevention.

## Figures and Tables

**Table 1:** The comparison of molecular weight and PI of RPL23A protein between *A. melanoleuca* and other four species.

	*Ailuropoda melanoleuca*	*Homo sapiens*	*Mus musculus*	*Rattus norvegicus*	*Macaca mulatta*
Molecular weight (kD)	17.7179	17.6919	17.6919	17.6919	17.6919
PI	11.16	11.16	11.16	11.16	11.16

**Table 2:** The differences in secondary structure of RPL23A protein between the *A. melanoleuca* and other four species.

Species	amino acid site					

	123	126	127	128	129	130
*A. melanoleuca*	H	H	H	H	H	H
*Homo sapiens*	H	H	H	H	H	H
*Mus musculus*	C	H	H	H	H	H
*Rattus norvegicus*	C	H	H	H	H	H
*Bos Taurus*	H	C	C	C	C	C
